# Medical Student Patient Outreach to Ensure Continuity of Care During the COVID-19 Pandemic

**DOI:** 10.1089/tmr.2020.0030

**Published:** 2021-02-19

**Authors:** Annika Belzer, Erin M. Yeagle, Lucille K. Kohlenberg, Muriel Solberg, Emily Gudbranson, Mariana Budge, Hannah M. Batchelor, Sarah E. Fitzpatrick, Anna Zhao, V. Diego Armengol, Samer F. Hassan, May Shum, Margaret Bia, Frank Bia, Nihar R. Desai, Peter A. Kahn

**Affiliations:** ^1^ Yale School of Medicine, New Haven, Connecticut, USA.; ^2^ Medical Scientist Training Program (MD-PhD), Yale School of Medicine, New Haven, Connecticut, USA.; ^3^ Department of Internal Medicine, Yale School of Medicine, New Haven, Connecticut, USA.

**Keywords:** telemedicine, medical education, COVID-19, pandemic, volunteer

## Abstract

**Background:**

In response to the COVID-19 pandemic, the Yale New Haven Health System began rescheduling nonurgent outpatient appointments as virtual visits in March 2020. While Yale New Haven Health expanded its telemedicine infrastructure to accommodate this shift, many appointments were delayed and patients faced considerable uncertainty.

**Objective:**

Medical students created the Medical Student Task Force (MSTF) to help ensure continuity of care by calling patients whose appointments were delayed during this transition to telemedicine.

**Methods:**

Eighty-five student volunteers called 3765 internal medicine patients with canceled appointments, completing screening for 2197 patients. Volunteers screened for health care needs, assessed preferences for future appointments, and offered emotional support and information about COVID-19. Urgent or emergent patient concerns were triaged and escalated to providers. In this analysis, we used a mixed-methods approach: call information and provider responses were analyzed quantitatively, and patient feedback was analyzed qualitatively via thematic analysis.

**Results:**

Ninety-one percent of patients screened found the MSTF calls helpful. Twenty-one percent of patients reported health concerns, with 1% reporting urgent concerns escalated to and addressed by providers. Themes of patient comments included gratitude for outreach and social contact, utility of calls, and well-wishes for health care workers.

**Conclusions:**

By calling patients whose appointments had been canceled during a rapid transition to telemedicine, the MSTF helped bridge a potential gap in care by offering patients communication with their care teams, information, and support. We propose that this model could be used in other care systems urgently transitioning to outpatient telemedicine, whether during ongoing outbreaks of COVID-19 or other public health emergencies.

## Introduction

In response to the COVID-19 pandemic, medical systems drastically reimagined and reframed their infrastructures for providing routine patient care, enabling a rapid expansion of virtual care in the outpatient setting. Previously adopted by specialties such as psychiatry and radiology, telemedicine offered a promising means to continue outpatient care while reducing the risk of infection, particularly at a time when personal protective equipment was scarce and relatively little was known about transmission of SARS-CoV-2.^1,2^ However, meeting the need for widespread telemedicine services posed an array of logistical and administrative challenges for both health care systems, which had to vastly expand their infrastructure for telemedicine, and providers, who had to adapt to conducting and billing for remote patient visits.^3^

Yale New Haven Health System began this process in early March 2020 by canceling upcoming nonessential in-person appointments and rescheduling them as virtual visits. Although facilitated by the health system's existing telemedicine infrastructure and its rapid mobilization of resources, this transition required time. As a result, a majority of patients were rescheduled for dates later than their original appointments, leading to delays until their next contact with a medical provider. In addition, some patients could not be contacted immediately for rescheduling, placing them at increased risk of lapses in care.

Shortly thereafter, the Yale School of Medicine suspended all in-person clinical activities for medical students, following recommendations from the Association of American Medical Colleges (AAMC).^4^ This created an unforeseen period of flexibility for clerkship students. In response, the authors, a group of students in their clerkship year, contacted leaders in the department of internal medicine to identify patient needs that students could help address. Many of these physicians expressed concern that patients, particularly those with chronic conditions, could face barriers to continuity of care while their appointments were rescheduled as virtual visits. At the time, most patients were not yet equipped with MyChart (Epic Systems, Verona, WI), the patient portal used at Yale New Haven Health for telemedicine visits and digital appointment scheduling. As a result, there was no established method available to rapidly contact patients *en masse* to screen for urgent health needs or reschedule virtual appointments that accommodated patients with limited access to computers or experience using technology.

The authors hypothesized that by calling these patients, medical students could apply their training to assess patients' health and medication needs during a time when patients were unable to see their providers as planned. Students temporarily suspended from their clerkships seemed particularly suited for this outreach, due to their clinical experience, technological literacy, and flexible schedules.

These conversations led to the organization of the Yale Medical Student Task Force (MSTF), a student-led patient outreach initiative. MSTF volunteers, primarily clerkship-year medical students, contacted patients whose in-person appointments had been canceled due to COVID-19 precautions to screen for health concerns. During these calls, volunteers also asked about preferences for future visits, provided basic information about COVID-19, and offered a source of support during an unprecedented and challenging time. In doing so, MSTF volunteers paralleled the efforts of volunteers across the world organizing remote outreach. Some of these efforts focused on aiding patients who were hospitalized during the pandemic,^5^ while others offered guidance to patients quarantining at home with COVID-19^6^ or company to older adults at risk for social isolation.^7,8^ Distinct from these endeavors, MSTF aimed to support patients with chronic medical needs during a time of unprecedented transition in outpatient care. Here, we describe the structure and evolution of this initiative and provide quantitative and qualitative analysis of its outcomes in addressing patient health concerns.

## Methods

### Organization of MSTF

MSTF was organized and led by 12 second-year medical students with support from internal medicine physicians, including two faculty advisors, one project manager, and a number of department-specific faculty consultants. Faculty advisors offered MSTF patient outreach services to Yale New Haven Hospital Internal Medicine departments, met with each department to tailor outreach and appoint faculty consultants, and were on-call to support volunteers when clinical concerns arose. The project manager was a third-year internal medicine resident who advised the MSTF leadership and obtained lists of patients within each department whose appointment(s) had been canceled due to crisis planning. Department-specific faculty consultants were on-call during days that their respective department's patients were contacted to respond to emergent and urgent patient needs and ensure appropriate follow-up.

**FIG. 1.**
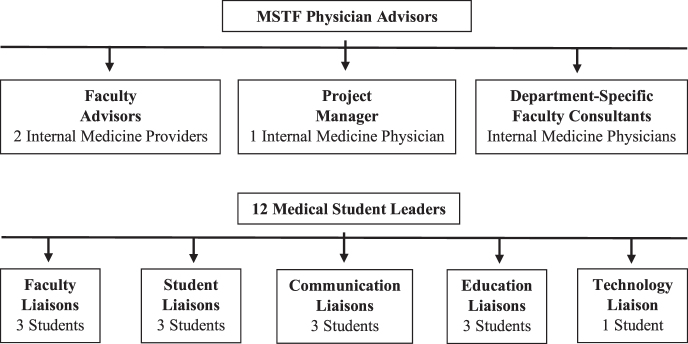
Organizational structure of MSTF (Medical Student Task Force).

The student leadership team was divided into five subcommittees, each with a distinct role in organizing the initiative and communicating with patients, providers, volunteers, and the Yale School of Medicine administration (Fig. 1). Faculty liaisons met with faculty and administrative leaders of each department to determine whether departments needed assistance in rescheduling patient appointments and to discuss which patients, if any, should be prioritized. Faculty liaisons developed patient outreach scripts and message templates for volunteers. They were on-call to triage urgent or emergent patient concerns reported by volunteers to department-specific faculty consultants and faculty advisors when provider action was needed. Student liaisons recruited and trained volunteers, coordinated outreach, and were on-call to address volunteers' logistical questions. The technology liaison coordinated technological aspects of patient outreach and was on-call to address technological problems. The medical students acting as communication liaisons were responsible for ensuring closed-loop communication between volunteers and health care providers. The education liaisons worked with the medical school administration and faculty leading the hospital's telehealth initiative to incorporate MSTF's patient outreach into an elective course at the Yale School of Medicine.

To ensure volunteers had clinical experience and comfort using Epic (Epic Systems), medical students were required to have completed at least one clinical clerkship to join MSTF. All volunteers participated in a live Zoom training (Zoom Video Communications, San Jose, CA) led by faculty advisors and student liaisons, during which each volunteer was provided with instructions on how to use a Doximity account to make calls. To accommodate varying schedules, volunteers were assigned patients to call based on their availability each week. The MSTF team ultimately included 73 volunteers in addition to the leadership team, for a total of 85 medical student volunteers, out of ∼300 eligible student volunteers. The names of all volunteers were sent to the Yale School of Medicine administration to ensure all involved students had clearance to use the Epic electronic medical record for patient outreach.

### Intervention

MSTF student leaders began by recruiting Yale New Haven Hospital Internal Medicine faculty to facilitate communication with each department, while simultaneously mobilizing Yale School of Medicine medical students as volunteers. Student leaders developed and disseminated a volunteer training packet with workflow guidelines, a patient outreach script, and an EPIC message template for contacting patients' providers. Patients from participating clinics were identified from lists of recently canceled appointments. Within 1 week of establishing the framework for MSTF, volunteers began contacting patients. Outreach began with patients of the department of endocrinology and expanded to include other departments who had not yet started the process of rescheduling patient appointments, including general cardiology, congestive heart failure, electrophysiology, benign hematology, malignant hematology, and sections within oncology.

The MSTF outreach used phone calls both to accommodate patients with less experience using technology and to enable real-time conversation and patient feedback. Outreach calls aimed to: (1) emotionally support patients during a time of isolation and uncertainty; (2) assess interest in and feasibility of future virtual appointments; (3) screen for urgent health care needs; and (4) offer basic information about the COVID-19 pandemic. Each call began with an open-ended prompt to give patients an opportunity to express anxieties related to COVID-19 and social isolation to a supportive listener (Supplementary Appendix SA1). Volunteers then asked patients whether their appointments had been rescheduled following cancellation. Patients rescheduled for a virtual appointment were asked if they preferred a phone or video visit. Patients who preferred video received brief setup instructions for Epic's MyChart app, the platform used at (Yale New Haven Health) for video visits with providers. A simple but important component of this conversation was to encourage patients to refrain from canceling upcoming appointments, as many patients had already done so due to the uncertainty and confusion about how health care clinics were following social distancing guidelines. This advice aimed to simplify rescheduling for each department and decrease the likelihood of delay until each patient's next contact with a provider.

To assess patients' health care needs, volunteers asked patients about their medication concerns and side effects, need for medication refills, and any changes in chronic health conditions. Patient-reported concerns were triaged by volunteers as emergent (necessitating a call to 911), urgent, or nonurgent. Issues were categorized as urgent if they required time-sensitive action, such as new concerning symptoms or medications that needed to be refilled within 3 days. Volunteers reported emergent and urgent issues to the student faculty liaisons, who in turn shared them with designated internal medicine physicians known as department-specific faculty consultants. Issues requiring less time-sensitive follow-up (nonurgent symptoms, prescription refills, medication questions) were communicated to patients' providers via Epic message.

To address the potential lack of reliable information regarding the COVID-19 pandemic, volunteers offered patients who asked questions or expressed concerns about the pandemic basic information from the Centers for Disease Control and Prevention (CDC) and (Yale New Haven) Hospital's COVID-19 websites.^9,10^ To assess the effectiveness of outreach, volunteers ended each call by asking patients whether they had found the outreach helpful. Answers were recorded as yes or no, with the option to include further description of patient comments if applicable.

Urgent or emergent patient health concerns were relayed by volunteers to faculty liaisons, who reviewed the information and passed it on to a department-specific faculty consultant. This consultant ensured that a member of the care team reached out to the patient directly. For nonurgent health concerns, volunteers sent EPIC messages to patients' providers using a provided template. To ensure follow-up, volunteers contacted their communication liaison if the provider did not acknowledge the message within 1 week. To help assess outcomes of each call and effectiveness of outreach, volunteers completed a postshift survey after each patient contact.

The postshift survey was updated using patient and volunteer comments, as well as department-specific requests. For example, the department of cardiology requested volunteers ask patients if they had discontinued an angiotensin-converting enzyme inhibitor (ACEi) or aldosterone receptor blocker (ARB) due to potential public misinformation. Volunteers also asked cardiology patients if they had begun taking azithromycin or hydroxychloroquine (Plaquenil) to treat COVID-19 due to the risk of arrhythmias and QT prolongation (Supplementary Appendix SA1). Volunteers then notified the on-call cardiology faculty consultant of all patients who answered yes to either question.

The process for volunteer calls was also adapted throughout the initiative, most substantially after completing calls for the pilot department, endocrinology. Based on provider recommendations, we limited Epic messages to providers whose patients' concerns necessitated follow-up and edited our call lists to include only patients rescheduled for >2 months from present or not rescheduled at all. To help address refill requests in a timely manner, volunteers were instructed to ask patients who required a refill if they had called their pharmacy or requested the refill through MyChart.

### Data collection

The study was approved by the Yale School of Medicine Institutional Review Board (IRB no. 2000028003). We performed a retrospective chart review of patients with one or more canceled appointments at a (Yale New Haven Health) clinic in the setting of the COVID-19 pandemic. Patients were included in the study if they had an outpatient appointment with endocrinology, cardiology, hematology, or oncology on or after March 1, 2020, and were referred to MSTF between March and May of 2020. Patients were excluded if they were not referred to MSTF, even if they had a canceled appointment during this time period.

Data were collected from volunteer postshift surveys and the medical record. When information about the nature of a patient's reported concern was not explicitly stated in the medical record or volunteer survey, as was the case for three patients, it was inferred from patient outreach or provider action documented in the medical record on the day of the MSTF call. Concerns were then grouped by type, with unique concerns categorized as “other.”

### Quantitative analysis

Data were analyzed using STATA Version 16 (STATA Corp., TX) and MATLAB R2019b (MathWorks, Natick, MA). Chi-square tests (using MATLAB's chi2cdf.m) were used to compare the prevalence of nonurgent concerns and medication refills between departments. Urgent concerns were not subjected to statistical comparison between departments, given the limited sample size.

### Thematic analysis

Thematic analysis was performed using a data-driven or inductive stepwise approach as outlined by Braun and Clarke.^11^ Two independent reviewers familiarized themselves with the data, generated initial coding of dating, reviewed and refined themes, and defined the prevalent themes. The two independent reviewers then discussed the themes they had identified from their coding with a third member of the study team to reach an agreement on prevalent themes.

## Results

Over 7 weeks, 85 volunteers called 3765 internal medicine patients, with each volunteer making a mean of 44.8 calls (standard deviation = 39.0, range 1–244). In total, 2197 patients were successfully contacted and surveyed for health concerns. Of these patients, 452 (21%) reported a health-related concern to the volunteer, such as a new symptom, medication issue, or need for refill. Ninety-one percent of all patients assessed, including those who reported no health concerns, found the outreach calls helpful. Prevalent themes of patient feedback identified in thematic analysis, along with illustrative comments for each, are detailed in Table 1. Major themes identified were as follows: (1) expression of appreciation for outreach from their care team; (2) utility of the call as a way to share concerns and/or learn information about the next steps in their medical care; (3) well-wishes for health care workers; and (4) an appreciation of social connection during a period of relative isolation. Many patients also described additional scheduling or medical details beyond the scope of the MSTF call script, which volunteers then conveyed to providers.

**Table 1.** Prevalent Themes in Patient Response to Medical Student Task Force Outreach

Theme	Example quotes
Outreach from health care team	“It is nice to know that you aren't being tossed to the wayside during this crisis, that people still care about you.”
	“I appreciate you guys touching base and I appreciate having access to my care team.”
Social interaction during isolation	“… [thank you] for taking the time to listen and [making me] feel less lonely.”
	“I live alone so it's always nice to talk to another person, the telephone interactions I'm having are very helpful to me now.”
Utility of outreach	“You've given me an opening to seek help, and now I finally feel ok.”
	“I forgot to ask my PCP this question so this call is perfect timing, I was worried I wouldn't be seen and figure this out”.
Gratitude for health care workers	“I appreciate what you and all the healthcare providers are doing to keep everyone safe.”
	“I am so thankful for all of you and the work you are doing on the front lines of this crisis.”

**FIG. 2.**
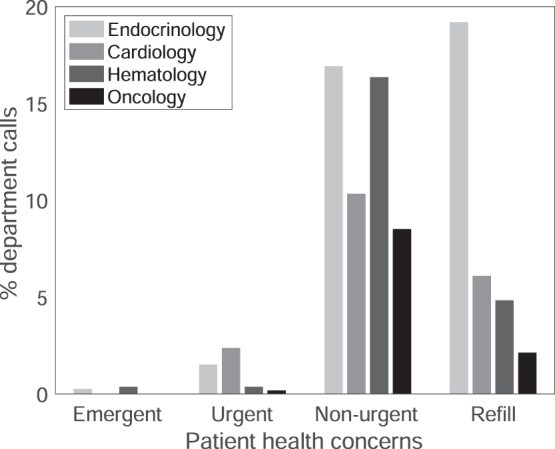
Patient-reported health concerns by department.

The majority of health concerns (92.6%) related by patients to MSTF volunteers were either nonurgent health concerns or requests for medication refills in which the patient had >3 days' supply (Fig. 2). Across departments, 259 patients (11.79%) had nonurgent health concerns other than medication refills, which were relayed to the patients' providers via EPIC. These were more prevalent in endocrinology and hematology than in cardiology or oncology (Fig. 2: across all departments *χ*^2^ = 23.23, *p* ≤ 0.0001). In addition, 160 patients (7.28%) requested a nonurgent medication refill. Nonurgent refill requests were most common in patients who had appointments with endocrinology (see Fig. 2: across all departments *χ*^2^ = 109.79, *p* < 0.0001).

Thirty-three patients (1.5% of total calls) reported urgent or emergent concerns to MSTF volunteers. These concerns were triaged by student faculty liaisons to a clinical provider (Table 2). Following triage, 22 of these patients (1.0% of calls) had their concerns addressed by physicians or nurses. Sixteen (73%) of these were in the department of cardiology, five (23%) were in endocrinology, one (4.5%) was in hematology, and one (4.5%) was in oncology. Concerns varied by department: the nature of each concern is outlined in Table 1. Two patients (one in hematology and one in endocrinology) reported emergent needs judged by the MSTF as necessitating a call to 911.

**Table 2.** Details of Patient Urgent or Emergent Concerns Leading to Provider Action

Department	Concern	No. of patients (% calls in department)
Endocrinology	Urgent refill	2 (0.0051)
	Hyperglycemia	2 (0.0051)
	Other	1 (0.0025)
Cardiology	Chest pain	6 (0.006)
	Urgent refill	5 (0.0052)
	Palpitations	4 (0.0041)
	COVID-related	2 (0.0021)
	Other	1 (0.0010)
Oncology	Other	1 (0.0018)

During calls for the department of cardiology, patients were asked about their medication use, specifically whether they were currently prescribed an ACEi or ARB, whether they had discontinued their ACEi or ARB, and whether they had recently initiated hydroxychloroquine or azithromycin to treat COVID-19. Full results for these questions are outlined in Table 3. Few patients reported medication change: five patients (2.3%) who reported they were currently prescribed an ACEi/ARB stated discontinuation of their ACEi/ARB and two patients (0.21%) reported new hydroxychloroquine or azithromycin use for treatment of COVID-19. A substantial minority (31%) were unsure if they were currently prescribed an ACEi/ARB, and a smaller proportion (7%) were unsure if they had recently initiated hydroxychloroquine or azithromycin.

**Table 3.** Medication Use Patterns in Cardiology Patients

	Yes	No	Unsure
Currently prescribed ACE/ARB (*n* = 968)	221 (22.8%)	448 (46.3%)	299 (30.9%)
Recently discontinued ACE/ARB (*n* = 221)	5 (2.3%)	216 (97.7%)	
Currently taking hydroxychloroquine or azithromycin (*n* = 968)	2 (0.21%)	887 (91.6%)	79 (8.2%)

ACE, angiotensin-converting enzyme; ARB, aldosterone receptor blocker.

## Discussion

By mobilizing a large group of medical student volunteers, the MSTF provided a novel approach to supporting outpatient care through the early transition to telehealth during the COVID-19 pandemic. During a period of rapid change and relative isolation for patients with chronic conditions, MSTF outreach calls offered patients a means to communicate their clinical and scheduling needs in real time, voice their concerns and anxieties about the pandemic to a supportive listener, and find concrete answers to basic questions about COVID-19. The initiative also empowered medical students to assist in alleviating the burden of the pandemic on clinical teams and patients. Although few patients reported urgent or emergent concerns, patient satisfaction with the MSTF initiative was high, with 91% of patients finding outreach helpful. Volunteers were able to complete screening for the majority (58.4%) of patients called. For patients who could not be contacted, MSTF leadership provided a list of names to each department.

Patient-reported concerns varied among departments, supporting the need for a department-specific approach to outreach. Nonurgent concerns were more common among patients with appointments in endocrinology and hematology, compared with oncology or cardiology. Meanwhile, nonurgent medication refill requests were by far most prevalent in patients seen by endocrinology, with nearly one in five patients in this group requesting a refill. While it is difficult to make comparisons given the evolution of the pandemic and the refinement of outreach protocols over time, this pattern may be related to the timing of outreach to endocrinology patients, who were in the first cohort of patients contacted by MSTF. If true, this suggests that early outreach is of critical importance in effectively addressing patient needs. Additional explanations may include differences between departments in appointment frequency, patient technological or medical literacy, and/or patients' initiative in reporting concerns to their providers. In future outreach, it may be helpful to prioritize departments whose patients are most likely to be impacted by the absence of in-person visits: those with less frequently scheduled visits and more limited access to providers or those whose patients have less technological or medical literacy.

Interestingly, cardiology patients contacted by MSTF reported few medication changes even during a period of conflicting news regarding medication use and risk for COVID-19, suggesting a mismatch between provider concerns and patient behavior in this department. While reassuring, interpretation of this finding is limited by the large proportion of patients called who were unsure of their current medications, as well as by limitations of the sample within a single department and health system.

The results of the MSTF initiative reaffirm the important role for telephone outreach in supporting remote medical care. Patients' positive response to MSTF calls, and volunteers' ability to identify patients with time-sensitive medical concerns and bring them to provider attention, underscore the value of telephone outreach as an adjunct to formal telehealth visits and the potential for phone calls to aid in remote management of both chronic and acute medical concerns. These observations suggest that telephone outreach merits continued support as a complement to outpatient telehealth.

Challenges in the development of MSTF included prioritization of patients, logistics of volunteer outreach, and consideration of patients not reachable by phone. In prioritizing departments, leadership considered when departments had reached out regarding MSTF services, department faculty availability to support MSTF, and department need. Of note, most departments were able to provide faculty to support MSTF, due to the increased number of physicians working from home at the time. Within departments, certain clinics were prioritized based on need per department request (for instance, oncology).

Organizing a large number of volunteers for calls was a significant undertaking, requiring student liaisons to commit multiple hours per day to volunteer support. Volunteers were initially assigned patient lists manually, but this process was expedited after student liaisons developed a MATLAB script to help automate the assignment of patients to each volunteer. To conserve volunteer resources, further outreach was not attempted for patients who could not be contacted after two calls. Instead, each department was provided with a list of these patients to ensure that the administrative teams were aware of patients not contacted during MSTF outreach.

The research element of this work is limited by several factors, including the inherent bias of volunteers asking for feedback on their own calls, which likely led to an overestimate of patient satisfaction. Although the majority of patients were successfully contacted by phone, a substantial minority (41.6%) were either not contacted or did not complete screening. These patients' concerns were not included in our analysis. As screening was completed in a single call, we were unable to evaluate changes in patient health concerns over time. Lastly, we did not examine the outcomes of the MSTF initiative on patient behavior or long-term health outcomes, although this may be a topic of interest for future research.

## Conclusion

In the setting of ongoing outbreaks of COVID-19, many health systems across the United States continue to reschedule in-person appointments as virtual visits, while medical schools continue to reimagine both in-person and remote clinical education. Although many health systems have substantially expanded their telemedicine services during the initial wave of COVID-19, some institutions, particularly those who have faced a lower local incidence of COVID-19 infection, have yet to develop an infrastructure to support widespread telemedicine and may face challenges in this transition. We propose that the MSTF outreach model could be adapted to serve patients of such institutions, both during the COVID-19 pandemic and in future public health crises requiring substantial restructuring of outpatient care.

Challenges to expanding the MSTF model include volunteer availability, as MSTF outreach was facilitated by a timely suspension of medical student clerkships. This may be addressed in academic medical centers by recruiting and training students in other health profession schools, as well as pre-clinical medical students, to expand the pool of available volunteers. Employment of the MSTF model outside of teaching hospitals would likely require either institutional or government funds to support volunteers with paid staff. Given the streamlined process of outreach with training guides, call scripts, and message templates, we believe that the framework of the MSTF model can be effectively used to serve a larger patient population.

## Supplementary Material

Supplemental data

## Supplementary Material

Supplementary Appendix SA1

## Abbreviations Used

AAMC: Association of American Medical Colleges
ACE: angiotensin-converting enzyme
ACEi: ACE inhibitor
ARB: aldosterone receptor blocker
CDC: Centers for Disease Control and Prevention
IRB: institutional review board
MSTF: Medical Student Task Force
