# Supplementary material for: Medical Student Patient Outreach to Ensure Continuity of Care During the COVID-19 Pandemic
Source: Telemed Rep. 2021 Feb 19;2(1):56–63. doi: 10.1089/tmr.2020.0030 (PMC9049823; doi:10.1089/tmr.2020.0030)
Supplement: Supplemental data [file Supp_AppendixSA1.docx]

*Phone* *Outreach* *Script* *for* *MSTF* *Volunteers*

Hello, is this [Patient Name Here]? My name is ________ and I am a medical student coordinator calling

on behalf of the [Institution] Department of Cardiology in regards to your recent appointment cancellation in

the setting of [Institution] Hospital’s COVID-19 precautions.

1. I know this is a stressful time for everyone, so I wanted to start by asking how you are holding up.

2. At this time, almost all outpatient visits with the [Institution] Department of Cardiology are being

rescheduled as virtual visits in order to protect the health of our patients and providers. Has anyone

reached out to you regarding rescheduling your Department of Cardiology visit?

a. *If* *YES:* Great. Please do not cancel this appointment. Were you told if this appointment is via

phone, video, or in-person?

b. *If* *NO:* Ok. A scheduler will be reaching out to you.

3. Our virtual visits are conducted via either phone or video call. If you would prefer a video visit, you

must have both a MyChart account and the MyChart app on your smartphone or tablet. Would you

prefer a video visit over a phone visit?

a. *If* *YES:* Ok. Do you currently have a [Institution] Hospital MyChart account and the

MyChart app?

i. *If* *YES:* Great.

ii. *If* *NO:* You can create an account at mychart.[Institution].org. If you require support,

please email mychartsupport@[Institution].org or call [phone number]. You can download

the MyChart app from the App store on either your smartphone or tablet.

4. Are there any medications that your cardiologist prescribes that you need urgently refilled?

a. *If* *YES:* What is the name and dosage of the medication? What is the name and address of

your preferred pharmacy? How many days do you have left on your current prescription?

i. Have you called your pharmacy about this prescription or requested a refill via

MyChart?

1. *If* *NO:* Please either call your pharmacy or request a refill via MyChart.

5. There has been some information in the news questioning whether two types of blood pressure

medications – angiotensin-converting enzyme (ACE) inhibitors and angiotensin receptor blockers

(ARBs) should be continued in the setting of COVID-19. Do you currently take an ACE inhibitor or an

ARB?

a. *If* *YES:* Have you recently discontinued your ACE inhibitor or ARB?

i. *If* *YES:* May I ask why?

1. *If* *due* *to* *fears* *regarding* *COVID-19:* [Institution] Cardiology recommends the

continuation of all of your blood pressure medications unless directed

otherwise by your cardiologist. Please restart taking this medication as

prescribed.

2. How many days have you been off this medication?

ii. *If* *NO:* Great. [Institution] Cardiology recommends the continuation of all of your blood

pressure medications unless directed otherwise by your cardiologist. Please

continue taking your medications as prescribed.

6. Some of the experimental treatments for COVID-19 can affect your heart. Are you taking either

azithromycin or hydroxychloroquine to treat COVID-19?

a. *If* *YES:* Ok. Please contact your cardiologist immediately to let them know. We will also pass

on this information to the on-call physician.

b. *If* *NO:* Ok. Please be aware that if any treatment for COVID-19 is offered to you, [Institution]

Cardiology recommends that you contact your cardiologist before beginning to take these

medications.

c. *To* *volunteers:* *If* *the* *patient* *reports* *a* *new* *prescription* *of* *azithromycin* *or*

*hydroxychloroquine,* *please* *contact* *the* *faculty* *liaison.*

7. I will be sending a private message to your cardiologist after this call regarding your healthcare

needs. Do you have any urgent questions about your cardiology medication(s) or medication side

effects that you would like to be relayed to your cardiologist?

8. Do you have any urgent questions about your cardiology health condition(s) that you would like to

be relayed to your cardiologist?

9. Have you experienced a decline in your cardiology health that you think needs to be more urgently

followed up virtually with a healthcare professional?

a. *If* *YES*: Could you please explain the change in your health?

i. *If* *you* *think* *your* *patient* *is* *experiencing* *an* *acute* *medical* *emergency*: I think you are

experiencing a true medical emergency that needs immediate attention. Please

hang up and call 911 now.

ii. *For* *all* *other* *concerns*: I will relay these concerns to your provider after I finish this

call.

b. *If* *NO:* We are glad to hear that you have no urgent concerns.

10. *If* *your* *patient* *has* *any* *question* *about* *COVID-19,* *please* *refer* *to* *the* *MSTF* *COVID-19* *Fact* *Sheet.* *You*

*may* *also* *refer* *them* *to* *the* *[INSTITUTION]* *COVID-19* *hotline,* *at* *[phone number].* *Please* *only*

*share* *general* *knowledge* *–* *NOT* *medical* *advice* *–* *regarding* *COVID-19.*

11. Before we finish this call, I wanted to ask: do you feel that this call was helpful to you?

12. Thank you for your patience as we try to keep our community safe during this difficult time. I will

make sure to update your healthcare provider about everything we discussed today.
